# Childhood obesity in the first 2000 days: A focus on primary health care in regional and rural Australia

**DOI:** 10.1111/ajr.13208

**Published:** 2025-02-05

**Authors:** Juliana M. Betts, Michelle Gooey, Alex Chung, Heather Craig, Heidi Bergmeier, Caroline Amirtharajah, Bernie Peacock, Sophie Ping, Kylie Rix, Stephanie Veal, Helen Skouteris

**Affiliations:** ^1^ Health and Social Care Unit, School of Public Health and Preventive Medicine Monash University and Monash Health Melbourne Victoria Australia; ^2^ Grampians Health Ballarat Victoria Australia; ^3^ City of Ballarat Ballarat Victoria Australia; ^4^ Victorian Maternal Child Health Coordinator's Group Melbourne Victoria Australia; ^5^ Ballarat Community Health Ballarat Victoria Australia; ^6^ Warwick Business School University of Warwick Coventry UK

**Keywords:** health services, infant, paediatrics, rural health, social determinants of health

## Abstract

**Introduction:**

Rates of childhood obesity have increased in regional and rural areas in Australia over the past two decades.

**Objective:**

To review the current literature to gain an understanding of (i) ways to improve access to primary health care in the first 2000 days, (ii) models of care for delivering healthy lifestyle advice in the first 2000 days and (iii) the development of partnerships between health and social care services in the first 2000 days in rural and regional settings.

**Design:**

Three literature reviews were undertaken. Results were limited to published, peer‐reviewed literature from the past 5 years (2017–2022).

**Findings:**

Access to care could be improved through the expansion of telemedicine, nurse roles and community health worker models. A range of organisational and leadership factors facilitated the integration of health and social care services in the first 2000 days in rural areas with evidence of resultant positive health impacts.

**Discussion:**

Telemedicine, the expansion of nursing roles and the implementation of a formalised community health worker model, may serve to improve access to primary health care for families in the first 2000 days; however, further research on particular models of care for delivering healthy lifestyle advice to rural and regional families is required.

**Conclusion:**

Policy‐makers should consider the interdependent nature of increasing access to care, establishing best practice models of care and strengthening local partnerships to prevent and manage childhood obesity in the first 2000 days in rural and regional areas.

**What is already known on this subject?**

- Rates of childhood obesity in regional and rural areas of Australia appear to have increased over the past two decades, despite improving in metropolitan areas.
- The social gradient in obesity prevalence necessitates a healthy equity approach to childhood obesity policy, which prioritises groups within the population who experience an inequitable burden of disease.
- The first 2000 days of life (the period from conception through to age 5 years) has been identified as a key developmental window of opportunity for targeted obesity prevention strategies.

**What this paper adds?**

- Policies to address childhood obesity in primary health care should consider the challenges of delivering obesity prevention and treatment in rural and regional settings, including barriers to accessing and receiving care.
- Telemedicine, the expansion of nursing roles and the implementation of a formalised community health worker model, may serve to improve access to primary health care for families in the first 2000 days; however, further research on particular models of care for delivering healthy lifestyle advice to rural and regional families is required.
- Strengthening local partnerships will likely result in improved health outcomes in the first 2000 days in rural settings.

## 1 INTRODUCTION

The first 2000 days of life (hereafter referred to as the first 2000 days), the period from conception through to age 5 years, has been identified as a key developmental window of opportunity for targeted obesity prevention strategies.^1^ Factors in the first 2000 days, such as parental Body Mass Index (BMI), in utero smoking exposure, breastfeeding, initiation of complementary feeding, screen‐time exposure and the nature of the infant–carer relationship, have been associated with a child's weight.^2^ Considering a quarter of Australian children aged 2–4 years' experience overweight or obesity,^3^ policies and interventions addressing childhood obesity in older, school‐aged children, may have less impact than those which aim to address the major predisposing factors from conception through to age five.^2^

Childhood obesity should be prioritised for policy action because it is generally preventable, has serious negative implications for child health and development and has long‐standing, possibly irreversible effects into adulthood.^4^, ^5^ The implementation of effective policies to prevent and manage childhood obesity in the first 2000 days has the potential to save billions of dollars when considering gains to productivity and consequent reductions in future health care costs.^6^

A health equity approach to childhood obesity policy, which prioritises groups within the population who experience an inequitable burden of disease, is required. Rural and regional communities, where social, demographic and environmental influences, are likely to perpetuate childhood obesity^7^ and where primary health care services are currently encountering unique sustainability challenges,^8^ are one such example. In Australia, rates of childhood obesity appear to have increased in regional and rural areas over the past two decades, despite decreasing rates in metropolitan areas.^7^ In the Australian context, access to high‐quality, affordable health services in rural and regional areas is becoming increasingly difficult, compounded by workforce shortages, and the increased costs associated with delivering medical care.^8^ Rural and regional populations should be prioritised for public health policy action, as noted in Australia's National Preventive Health Strategy 2021–2030.^9^

An integrated and well‐resourced primary health care system has been emphasised as a means of achieving universal health coverage and delivering ‘health for all’ in accordance with the United Nations Sustainable Development Agenda.^10^ A comprehensive model of primary health care, which is defined by the World Health Organization as a ‘whole‐of‐society approach’ that considers the broader social, economic and commercial determinants of health, is well‐placed to address childhood obesity in the first 2000 days.^11^ However Australia's primary health care system, which has tended to focus on the provision of medical services, requires significant reform, particularly to ensure that inequity gaps are not widened.^12^

Evidence demonstrates that collaborations between researchers and policy and practice stakeholders can enhance the relevance and application of evidence in policy and practice.^13^ We therefore collaborated with a range of primary health care stakeholders (who are coauthors on this paper) to identify key areas for policy reform to address childhood obesity in the first 2000 days in the rural and regional Australian primary health care context. Key areas for policy reform identified through discussions with primary health care professionals, formed the basis of three literature reviews to determine evidence‐based solutions to inform future obesity policy development.

Accordingly, this comprehensive series of evidence reviews aimed to identify:

1. Methods for improving access to primary health care in rural and regional areas in the first 2000 days;
2. Models of care for delivering healthy lifestyle advice in the first 2000 days in regional and rural settings; and
3. Examples of effective partnerships between health and other organisations in the first 2000 days.

## 2 METHODS

Three literature reviews were undertaken. Each review adhered to a study protocol, which was constructed prior to undertaking the search (Data S1). The databases Medline, Global Health and PsycINFO were searched in the OVID platform, using terms relevant to each policy theme. Results were limited to the peer‐reviewed literature from the past 5 years (2017–2022).

Results were screened for inclusion by a senior researcher (HC) using the software Covidence.^14^ Data were extracted on Country of Origin, Study Design and Key Findings. Quality was formally assessed using the Joanna Briggs Institute (JBI) Quality Appraisal tool relevant to the study design. For study quality to be considered high, all elements of the JBI checklist must have been met. For study quality to be considered moderate, at least 60% of the JBI checklist was met. For study quality to be considered low, fewer than 60% of the JBI checklist was met.

### 2.1 Ethics

Formal ethics approval was not sought for this study.

## 3 RESULTS

### 3.1 Review 1: Improving access to primary health care in rural and regional areas in the first 2000 days

A literature review of international evidence retrieved an initial 4344 records, of which 21 underwent full‐text review. Seven studies were deemed eligible for inclusion and are summarised in Table 1 (see Data S2 for PRISMA flow diagram). Three studies explored telemedicine interventions,^15^, ^16^, ^17^ one study pertained to a care navigator role,^18^ two studies evaluated a dental program utilising lay community health workers^19^, ^20^ and one study described the case files of home‐visiting nurses.^21^ Quality assessments revealed that all of the included studies were of moderate quality (see Data S3 for quality appraisal checklists).

**TABLE 1:** Features of included studies in literature review 1: Improving access to primary health care in rural and regional areas in the first 2000 days.

Citation	Country	Study design	Primary mode of improving access	Key findings	Quality appraisal (low, moderate or high)^a^
Demirci et al. (2019)^13^	USA	Qualitative (semi‐structured interviews)	Telemedicine	Tele‐lactation was convenient and efficient for women in rural areas. Barriers to use included maternal reluctance to conduct video calls with an unknown provider, preference for community‐based breastfeeding resources and technical issues (e.g. limited WiFi in rural areas)	Moderate
Kapinos et al. (2019)^14^	USA	Descriptive (case series) evaluation	Telemedicine	Compared with non‐users, participants who used tele‐lactation were more likely to be working within the first 12 weeks' postpartum compared with others, were less likely to have prior breastfeeding experience and were less likely to have breastfed exclusively prior to hospital discharge. Most users (91%) expressed satisfaction with the help received via the tele‐lactation consultation	Moderate
Kirby et al. (2021)^16^	Australia	Qualitative (observation and interviews)	Care navigator (nurse)	The program was perceived to improve client families' lives in relation to children's health and other family health and social issues. Trust in the care navigator was the most important factor for parents to join and engage with the program	Moderate
Luscombe et al. (2021)^15^	Australia	Qualitative	Telemedicine	The virtual feeding clinic was perceived as convenient for families requiring a specialised service and led to increased attendance. Other perceived benefits of the program included enhanced continuity of care and experiential learning and networking for clinicians. Challenges of providing the service included the limited hours of operation, ongoing funding, and some physical and administrative challenges	Moderate
Mathu‐Muju et al., 2017^17^	Canada	Qualitative	Lay community health workers	The local, community‐based nature of the initiative was viewed as essential to its success in improving access to preventive dental services and improving children and caregivers' oral health knowledge and behaviours	Moderate
Mathu‐Muju et al. (2018)^18^	Canada	Case series evaluation	Lay community health workers	Community health workers were beneficial in promoting program enrolment, as well as facilitating and augmenting the delivery of preventive dental services	Moderate
Wideman et al. (2020)^19^	USA	Qualitative	Nurse home visiting program	Agencies serving rural areas should allocate resources and adapt training to support nurses based on unique community profiles	Moderate

^a^ Quality appraisal based on JBI quality appraisal tool—full results available in Appendix S1.

#### 3.1.1 Telemedicine

Among the three studies evaluating telemedicine interventions in rural areas, two were for breastfeeding support^15^, ^16^ and one was for the multidisciplinary management of paediatric feeding issues.^17^ While the two breastfeeding support interventions were identical phone‐based applications for video consultation with a Lactation Consultant, the virtual feeding clinic provided a ‘hub and spoke’ model of care with families presenting to a consultation room with videoconferencing capability.

All three studies highlighted the access benefits of telemedicine to rural populations where it has the potential to overcome health service shortages. The breastfeeding support interventions were perceived to overcome particular issues for mothers with infants, who were able to access tele‐lactation support from their homes 24 hours per day.^15^, ^16^ Similarly, the virtual paediatric feeding clinic overcame the challenges associated with travelling long distances with children, particularly for those who may have special needs. There was also some evidence that the tele‐lactation support had reasonable uptake among women of lower socio‐economic status.^15^, ^16^

The benefits of telemedicine were perceived to extend beyond access for individual patients, to the upskilling and empowerment of fellow health professionals who sat in on the telehealth consultations.^15^, ^17^

All three of the included studies on telemedicine noted technical issues as a barrier to implementation, which included trouble logging in and WiFi coverage; however, these issues were considered to affect a minority of consultations.^15^, ^16^, ^17^ The ongoing availability of funding and physical resources was considered a challenge for the paediatric feeding clinic, and it was only offered during business hours one day per week.^17^ Apprehension about telemedicine, such as discomfort with talking to a stranger over video, was noted to be a concern among mothers accessing telelaction.^18^ For the virtual feeding clinic, this apprehension was somewhat counteracted by the fact that an initial in‐person visit at the hub site was undertaken, which was perceived to have facilitated rapport.^17^

#### 3.1.2 Nurse‐led programs

Two studies analysed the role of a clinical nurse in providing additional support to rural families in need.^18^, ^21^ One was a home‐visiting health nurse and the other was a ‘care navigator’. Both studies highlighted the many barriers to accessing health care in rural areas, which extended beyond merely the provision of services, to incorporate the unique geographical, social and cultural determinants at play. For example, Wideman et al. demonstrated that a lack of social support in the form of childcare hampered a mother's adherence to psychiatric medications,^21^ and Kirby et al. highlighted that previous experiences of discrimination limited families' access to health and social care services.^18^ Both studies emphasised the ability of the nurse to understand the broader ‘whole picture’ of the family within their context, and advocate for access to a range of health and social services, including vital material needs such as housing and food.

Enablers of the nursing programs included good working relationships between the nurse and other local health and social services, which facilitated referrals. In particular, Kirby et al. noted the nurse's ability to access and understand medical records as a key facilitator for the development of care plans and appropriate referrals.^18^ Other important features of the nurse included tenacity, commitment, a non‐judgemental approach, knowledge of the town and a profile within the town.

Barriers to the nursing programs included staff turnover and staff shortages, which hampered interprofessional collaboration and working relationships.^18^ Uncertainty regarding ongoing funding was also a barrier that was perceived to amplify mistrust in the community.^18^ For the home‐nursing program, telephone access and transportation were significant barriers for both clients accessing services and nurses' ability to deliver support.^21^

#### 3.1.3 Lay community health workers

The two studies included in this review, which evaluated the role of lay community health workers in improving primary health care access, pertained to the Canadian Children's Oral Health Initiative (COHI), a federally funded community‐based preventive dental program for Indigenous communities.^19^, ^20^ The program is delivered by COHI aides who are lay community health workers, residing in the community and trained to work with dental therapists to deliver screening and preventive dental advice for children aged 0–7 years.

Interviews with parents and caregivers of child participants emphasised the local, community‐based nature of the program as a key element of its success, enabling for prompt and easily accessible preventive treatment of early dental disease.^19^ In particular, the COHI aide facilitated continuity of care where the dental health workforce is often transient. Facilitated access to early prevention and intervention was noted to have positive impacts for the community by way of the perception of fewer families needing more intensive surgical dental management outside the community, although this measure was not quantifiably analysed.

In an analysis on the effect of the availability of the community health worker on access to preventive dental services among 25 communities participating in the COHI program, it was demonstrated that communities with uninterrupted access to a community health worker tended to have the highest rates of enrolment in the dental program and therefore the highest rates of service delivery.^20^ By contrast, communities with only sporadic access to a COHI aide tended to have low rates of enrolment and service delivery.

### 3.2 Review 2: Models of care for delivering healthy lifestyle advice in the first 2000 days

Results from the literature review exploring the models of care for delivering healthy lifestyle advice in the first 2000 days in rural and regional settings, returned an initial six studies for inclusion, from 6272 identified records. Three studies were removed from the results as they pertained to the telehealth interventions, which had been captured in Review 1.^15^, ^16^, ^17^ Features of the remaining three included studies are summarised in Table 2 (see Data S2 for PRISMA flow diagram). Quality assessments demonstrated that the studies were of moderate quality (Data S3).

**TABLE 2:** Features of included studies in literature review 2: Models of care for delivering healthy lifestyle advice in the first 2000 days.

Citation	Country	Study design	Model of care for delivering healthy lifestyle advice in the first 2000 days	Key findings	Quality appraisal (low, moderate or high)^a^
Ahlers‐Schmidt et al. (2019)^20^	USA	Pre‐ and post‐intervention surveys	Instructor‐led community baby showers	Significant improvements in mothers' intention to follow Safe Sleep Guidelines and breastfeed were noted following the baby shower. 95% of participants reported they were satisfied or very satisfied with the shower	Moderate
Ekambareshwar et al. (2021)^21^	Australia	Qualitative process evaluation	Telephone call and text message‐based intervention	Successful program implementation was attributed to contextual factors: strong support by the host organisation, good project leadership, clear communication, collaborative internal and external partnerships, and intervention provision by experienced nurses Remote delivery was convenient to program participants and participants were able to resolve other personal concerns Because of their capacity to influence policy decisions, the absence of policy‐makers at project meetings was a shortcoming	Moderate
Johnson et al. (2017)^22^	New Zealand	Qualitative cross‐sectional survey	Breastfeeding peer counsellor program	Participants who underwent training to become peer counsellors tended to report an increase in breastfeeding knowledge, confidence and acceptance. They also reported a personal satisfaction with having undertaken the training and the perceived benefit it has had on the community	Moderate

^a^ Quality appraisal based on JBI quality appraisal tool—full results available in Appendix S1.

The three models of care identified for delivering healthy lifestyle advice in the first 2000 days in rural areas were as follows: an instructor‐led community baby shower, which focused on safe sleep and the prevention of sudden infant death syndrome (SIDS); a telehealth program offering text‐message and telephone‐based healthy lifestyle advice to mothers from the third trimester until their child turned 2 years of age; and a breastfeeding peer counsellor program. None of the three included studies included long‐term follow‐up for changes in health outcomes. Ahlers‐Shmidt et al.^22^ did demonstrate significant improvements in participating mothers' knowledge around safe sleeping and intention to breastfeed; however, follow‐up to assess whether these changes translated to improved health outcomes by way of increased breastfeeding rates or decreased smoking rates was not part of the study.

None of the included studies demonstrated models of care for delivering healthy lifestyle advice in the first 2000 days involving a medical practitioner. Alternative models for delivering this advice were highly trained community and child health nurses^23^ and trained community members.^22^, ^24^ Similarly, none of the programs involved end‐users' face‐to‐face attendance at a health clinic, despite health clinics overseeing the programs and their implementation.

### 3.3 Review 3: Building partnerships with other health and social care organisations in the first 2000 days

Results from the literature review of international evidence (Table 3) demonstrated examples of how primary health care services can partner with other health and social care organisations to promote a more comprehensive model of primary health care in the first 2000 days. Of the 9916 records screened, 12 studies were deemed eligible and included in the review^25^, ^26^, ^27^, ^28^, ^29^, ^30^, ^31^, ^32^, ^33^, ^34^, ^35^, ^36^ (see Data S2 for PRISMA flow diagram). All of the included studies were evaluations of existing health programs. Five of the 12 studies used qualitative methods,^29^, ^31^, ^32^, ^35^, ^36^ four studies used quantitative data from surveys or health records,^25^, ^26^, ^27^, ^30^ one study used mixed methods,^28^ one study was a randomised clinical trial,^34^ and one study was a case–control study.^33^ Most of the health programs being evaluated were targeted towards parents and children with higher levels of socio‐economic disadvantage.^25^, ^26^, ^27^, ^29^, ^31^, ^32^, ^35^, ^36^ Quality assessments demonstrated that the majority of studies were considered to be of moderate quality (see Data S3 for quality appraisal checklists).

**TABLE 3:** Features of included studies in literature review 3: Building partnerships with other health and social care organisations in the first 2000 days.

Citation	Country	Study design	Nature and goal of health partnership	Key findings	Quality appraisal (low, moderate or high)^a^
Brown et al. (2020)^23^	UK	Repeated measures case series	Statutory health services (e.g. midwives) and local civic organisations aiming to improve maternal health	Improvements in anxiety and depression scores among mothers, improved health literacy, positive changes in network size and social capital	Moderate
Corley et al. (2022)^24^	USA	Cross‐sectional survey	Primary health service and faith‐based organisations aiming to increase child influenza vaccination rates	600 paediatric influenza vaccinations were administered between 2016 and 2019. 1162 participants underwent health screening, with 37.9% having abnormal BMI, 52.5% having abnormal dentition and 29.7% having abnormal vision	Low
Gold et al. (2018)^25^	USA	Retrospective audit	Embedding dental professionals within the Women, Infants and Children (WIC) program to improve access to dental services	59.1% of pregnant women had untreated decay. 71.2% of pregnant women had unmet dental care needs. 34.8% of 3‐year‐olds had dental caries	Moderate
Hargreaves et al. (2017)^26^	USA	Mixed methods evaluation	Primary care, public health and community‐based organisations aiming to prevent and treat child obesity	The level of engagement with the program varied between teams. Those with pre‐existing relationships were more engaged. Member turnaround and clinician time were barriers to effective engagement. Levels of collaboration improved over time, and one‐third of teams had developed sustainability plans	Moderate
Kay et al. (2019)^27^	UK	Qualitative program evaluation	Attaching an oral health worker to the Family Nurse Partnership program to improve dental health among infants and children	Seven key themes influenced parents' ability and willingness to accept and interact with an oral health intervention: personal experiences of oral health, oral health knowledge, visiting dental services, timing of visit (co‐incidence of teething), what advice and support was wanted (brushing and how to establish routine), family norms and the importance of a trusted relationship with the nurse practitioner. Autonomy and degree of control over the stability of their environment, lack of knowledge and conflicting information were perceived barriers to the delivery of the intervention	Moderate
Olson et al. (2018)^28^	Canada	Cross‐sectional survey	Linkage between a range of services including acute care hospitals, rural facilities, midwives, doctors, public health nurses and community organisations to bridge care between discharge from hospital and the needs of mothers and infants in the early post‐partum period	82% of women (*n* = 403) were very satisfied with their experience and more than 86% agreed/strongly agreed that: Nurses answered their questions and addressed their concerns, the nurses visited an adequate number of times, the nurse supported their feeding plan, and left them feeling comfortable in their ability to care for their newborn	Moderate
Pawloski et al. (2022)^29^	USA	Qualitative program evaluation	Medical‐dental integration program to improve access to dental care and reduce childhood caries	The program was feasible and acceptable (with key considerations being setting—including charting and service integration, progressive leadership, and effective communication—integrated electronic medical records). Implementation had structural, systemic and individual behaviour barriers	Moderate
Rehmus et al. (2021)^30^	Canada	Descriptive case study	Paediatric dermatology integrated within a social paediatric program (primary health care nurses working with community services and specialised paediatric physicians) aiming to improve dermatology access and health outcomes in marginalised communities	The following elements were central to the success of the program: partnerships, bridging trust, knowledge sharing, empowerment, consistency and flexibility	Low
Salomonsson (2021)^31^	Sweden	Case–control	Psychotherapists incorporated into a nurse‐led child health care centre aiming to improve parent mental health and infant social–emotional functioning	Significant improvement in mothers' anxiety and depression scores between baseline and follow‐up. Fewer concerns among mothers regarding their infants' social–emotional functioning following therapy	Moderate
Taveras et al. (2017)^32^	USA	Randomised clinical trial	Primary care clinicians working with community health coaches to reduce childhood obesity and improve health‐related quality of life and parental resource empowerment	At 1 year, BMI *z*‐scores improved for both intervention arms (enhanced primary care plus health coaching) (BMI *z*‐score change −0.09); enhanced primary care only (BMI *z*‐score change −0.06)). There was no significant difference between the two intervention arms for any outcomes. Child health‐related quality of life improved for the enhanced primary care plus health coaching group only. Both intervention arms led to improved parental resource empowerment	High
Williams et al. (2022)^33^	USA	Qualitative case study	Nurse‐family partnership community program collaborating with Primary Care Providers to improve families' experience of care and meet the broader social needs of families	The population served by the program were considered to be underserved and publicly insured. Collaboration started at the referral process and continued throughout service co‐ordination. Mutual awareness, cooperation and collaboration were needed to service high‐needs families	Low
Williams et al. (2022)^34^	USA	Qualitative	Nurse home visitors, health care providers and community support services aiming to improve the health of families experiencing adversities	Facilitating factors for collaboration included relational (e.g. shared values), organisational (e.g. leadership) and structural (e.g. policy and system integration) elements	Moderate

^a^ Quality appraisal based on JBI quality appraisal tool—full results available in Data S3.

Key themes emerging from the included studies related to organisational and leadership factors facilitating integration of health and social care services in the first 2000 days, barriers to service integration and benefits of integrated services (qualitative and quantitative outcomes).

#### 3.3.1 Organisational and leadership factors facilitating integration of health and social care services in the first 2000 days

Having leaders within the organisations, who supported the integration of services, tended to be perceived as crucial to successful partnership and program delivery.^28^, ^31^ The importance of buy‐in from all parties and, in particular, a strong ‘local champion’, was reiterated as a necessary element to the success of service integration.^28^, ^31^, ^35^ Local champions tended to be health care workers (e.g. midwives, social workers or others) who were committed to the partnership, and who ensured relationships between service providers were maintained.

Having a shared mission between services was considered an essential factor for integration.^28^, ^32^, ^35^ This enabled the different service providers to work towards a common goal and united them in their value‐based approach. For example, in Williams et al.'s qualitative review of a collaboration between nurse home visitors, health care providers and community support services in the United States, staff reported that mission congruence in the form of a strength‐based and client‐centred approach resulted in more frequent collaboration.^36^

Community engagement was another factor critical to the success of several health programs. Examples of effective community engagement were a community‐designed and led program for mothers,^25^ employment of members of the target population for service delivery^31^ and partnerships outside the health sector such as with faith‐based organisations.^26^

Other organisational factors that facilitated service integration tended to focus on enhancing communication between services. This was achieved through shared physical working spaces,^30^, ^31^, ^32^ shared health records and information technology (IT) systems^28^, ^31^ and regular opportunities for interaction between staff such as frequent community meetings^32^, ^35^ and joint training exercises.^32^, ^35^

#### 3.3.2 Barriers to service integration

A number of barriers to service integration were identified among the included studies. These included staff turnover,^35^ lack of funding,^31^, ^35^ lack of formal communication channels hampered by inadequate inter‐professional feedback loops and the absence of shared electronic health records,^35^ as well as instances where consumers received conflicting information.^29^

#### 3.3.3 Benefits of integration between health and social care organisations

The perceived and objective benefits of the collaboration between health services were outlined in the evaluations. Objective improvements in health outcomes were demonstrated for maternal mental health,^25^, ^33^ child health‐related quality of life,^34^ parental health literacy^25^ and parental social capital.^25^

Staff perceived benefits of service integration included greater access to care and services,^31^, ^35^ improved clinical outcomes for patients^31^, ^32^ and the ability of programs to foster trusting relationships with priority community groups who have historically experienced negative encounters with health care services.^31^, ^32^

There was also some evidence of consumer satisfaction with programs,^25^, ^29^, ^30^, ^34^ although most evaluations were limited to staff experiences of the program.

## 4 DISCUSSION

This research sought to understand what evidence‐based solutions currently exist to inform future policies that aim to improve the delivery of primary health care in the first 2000 days for the prevention and management of childhood obesity. It focused on three key themes:

1. Equity of primary health care access in the first 2000 days;
2. Models of care for delivering healthy lifestyle advice in the first 2000 days; and
3. The development of local partnerships to achieve a more comprehensive model of primary health in the first 2000 days.

**FIGURE 1:**
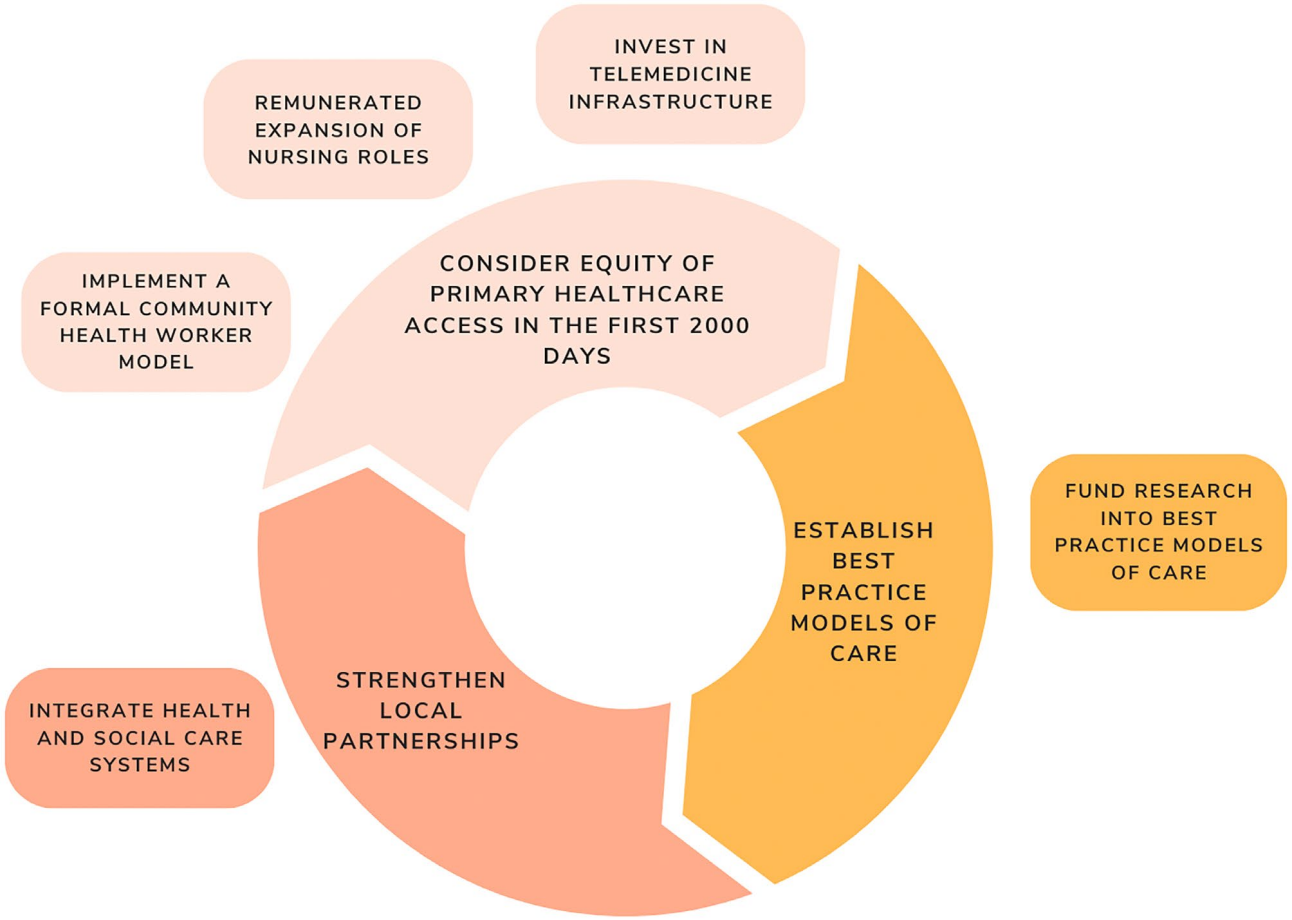
Addressing childhood obesity in the first 2000 days in rural and regional Australia; implications for policy‐makers.

The evidence highlights key policy recommendations, which are depicted in Figure 1 and discussed below. Increasing access to primary health care providers for priority populations is necessary to then initiate best practice models of obesity prevention and treatment to those most in need. Models of care that consider complex social needs (in line with known risk factors^2^ and the social gradient of childhood obesity prevalence^37^) will benefit from referral pathways, which are established through the strengthening of local partnerships. In turn, strong local partnerships are likely to enhance equity of primary health care access in the first 2000 days as social organisations are often the first point of engagement for people seeking assistance with their basic needs—food, housing and social connection.

### 4.1 Equity of primary health care access in the first 2000 days

Access to health care is considered a fundamental human right, yet close to 10% of people living outside major cities had no access to any primary health care service within a 60‐min drive time.^8^ Geographical considerations are but one element of health care access, and intersections with other social and cultural health determinants including poverty and racism, are known to impact one's ability to access health and social care.^38^ In the Australian context, the sustainability of the primary health care sector is in crisis, compounded by burnout, chronic workforce shortage and under‐funding.^8^

Access to primary health care services could be improved in the first 2000 days in rural and regional areas through an investment in telemedical infrastructure, the remunerated expansion of nursing roles and implementation of a formal community health worker (CHW) model.

#### 4.1.1 Invest in telemedicine infrastructure

Telemedicine refers to the provision and delivery of health care services using information and communication technologies.^39^ It includes the exchange of information for diagnosis, treatment and prevention of disease using a broad variety of modalities. In the context of the first 2000 days, telemedicine has additional accessibility benefits for rural parents who may face barriers to accessing traditional face‐to‐face health consultations, owing to both geographical and social factors. The ability to access specialised services in this way (such as lactation consultants, dieticians and speech pathologists) has the potential to improve child health and development in the first 2000 days by providing parents with the support they need to breastfeed and address early feeding issues—known determinants of early child obesity.^2^

The COVID‐19 pandemic catalysed the implementation of telemedicine services throughout the world, yet telemedicine has played a role in the rural and remote Australian health context for decades. Despite the potential advantages for increasing accessibility, it is essential that implementation of telemedicine in the first 2000 days pays careful attention to limitations of this model, particularly so as to not exacerbate health inequities. Potential barriers to the use of telemedicine in this context include a lack of technical infrastructure (including internet) in rural areas, concerns about privacy and confidentiality and a loss of non‐verbal communication, which is particularly pertinent to the discussion of sensitive issues (including weight management).^40^ The widespread uptake of smartphone technology, the rollout of the National Broadband Network as well as the adoption of a hub and spoke approach^17^ may serve to counteract some of these limitations.

#### 4.1.2 Remunerated expansion of nursing roles

Nurses' abilities to transcend the boundaries between health and social care have been highlighted in our review as a means of facilitating access to health care, particularly for those most vulnerable to the social determinants of health.^18^, ^21^ Further evidence for the success of this model is demonstrated through the Maternal and Child Health nurse program in the state of Victoria, which consistently demonstrates high participation throughout rural and regional areas, particularly in the first year of life.^41^ Care navigation is a central element of this role, because it recognises and addresses the myriad of competing priorities for families in need—such as housing and food security.

#### 4.1.3 Implement a formal community health worker model

The CHW model is a well‐recognised feature of many global health programs aiming to increase universal health coverage in areas with limited access to formally trained medical personnel. In the Australian context, Aboriginal Health Workers (AHWs) have provided this model of care through the Aboriginal Community‐Controlled Health Organisation (ACCHO) sector for several decades and have demonstrated efficacy in terms of improving cultural safety of, and access to, primary health care services.^42^ This formalised model, applied more broadly to other priority population groups within Australia, may serve to enhance equity of access to primary health care while also enhancing health literacy and community development, particularly in areas with health workforce shortages.^43^

### 4.2 Models of care for delivering healthy lifestyle advice in the first 2000 days

#### 4.2.1 Fund research into best practice models of care

Evidence from the literature indicated that telehealth and community health worker approaches may provide innovative models of care for delivering healthy lifestyle advice in the first 2000 days in rural and regional areas. However, the included studies did not investigate long‐term health outcomes in terms of improved behaviours in the first 2000 days. Telemedical approaches to the treatment of childhood obesity^44^ and the improvement of breastfeeding rates^45^ have demonstrated effectiveness in systematic reviews, yet their applicability to rural and regional settings remains uncertain.

Evidence on the effectiveness of community health worker models for paediatric weight management is scarce; however, a systematic review assessing the effectiveness of home‐based paediatric weight management services demonstrated that programs utilising professional staff (e.g. dieticians) tended to be more successful than programs adopting paraprofessional or community‐based staff.^46^ This may demonstrate the requirement for more rigorous training of community health workers or that information from trained health professionals is preferred.

An Australian‐based randomised control trial investigating the effects of a home‐based nursing program for pregnant women experiencing adversity demonstrated effectiveness on a range of developmental indicators, which may have relevance to obesity prevention (including more regular child bedtimes, warm parenting and variety of home experiences).^47^ Further investigation into home‐based and other models of care focusing on childhood obesity prevention and treatment in the first 2000 days in rural and regional areas is required.

### 4.3 The development of local partnerships

Primary health care sits within a broader model of primary care, which provides for the community's health needs through addressing the social and environmental determinants of health and empowering individuals, families and communities to live healthy lives.^11^

Many social determinants of health are known to impact on obesity risk factors in the first 2000 days. For example, women experiencing domestic violence during pregnancy demonstrated lower rates of breastfeeding self‐efficacy than those who did not.^48^ Similarly, children living in rural and disadvantaged areas are more likely to consume sugar‐sweetened beverages than those living in cities.^49^ Non‐medical factors influencing health and development in the first 2000 days must be considered through better integration of health and social systems in order to address the needs of those most at risk of childhood obesity.

#### 4.3.1 Integration of health and social care systems

Integration of health and social care systems can have positive outcomes in the first 2000 days such as improved maternal mental health, social capital and health literacy.^25^, ^33^ Moreover, we provided evidence on how health and social care organisations can work together to improve health outcomes in the first 2000 days. Ingredients for success included the following: strong organisational leadership,^28^, ^31^ local champions,^28^, ^31^, ^35^ mission congruence between organisations,^28^, ^32^, ^35^ community engagement^25^, ^26^, ^31^ and methods for enhanced cross‐disciplinary communication and training including co‐location of services^30^, ^31^, ^32^ and shared information management systems.^28^, ^31^

### 4.4 Strengths and limitations

This is a unique body of research, which has adopted a collaborative, stakeholder‐informed approach with key personnel involved in the Australian primary health care and policy context to understand policy needs and summarise evidence‐based policy solutions to addressing childhood obesity and its risk factors in the first 2000 days. The findings have numerous policy implications (Figure 1) and highlight the need to address equity.

It is recognised that the literature reviews undertaken are unlikely to have captured all available research on these topics, and it is possible that other interventions and strategies have not been included. However, this research did not set out to provide an exhaustive list of all possible policy solutions as our goal was to provide a preliminary assessment of the types of issues that policy‐makers should consider to address the problem of child overweight and obesity in the first 2000 days in rural and regional settings.

A limitation of this work is that it was not informed by a consumer with lived experience of being a rural‐dwelling parent to a child with obesity. Policy‐makers looking to address childhood obesity in the first 2000 days should make a concerted effort to engage with a broad range of stakeholders including end users and consumers of obesity prevention programs.

In conclusion, policies to address childhood obesity in the first 2000 days in rural and regional areas must consider options for improving access to health care in these settings, the need for further research into evidence‐based models of care in rural and regional areas and the importance of local partnerships for health and social care integration to ensure equity gaps in obesity outcomes do not continue to widen.

## AUTHOR CONTRIBUTIONS

**Juliana M. Betts:** Conceptualization; methodology; investigation; writing – original draft; writing – review and editing; visualization; formal analysis. **Michelle Gooey:** Conceptualization; methodology; writing – original draft; writing – review and editing; visualization. **Alex Chung:** Conceptualization; methodology; writing – original draft; writing – review and editing; visualization. **Heather Craig:** Investigation; methodology; writing – original draft. **Heidi Bergmeier:** Conceptualization; writing – review and editing. **Caroline Amirtharajah:** Conceptualization; writing – review and editing. **Bernie Peacock:** Conceptualization; writing – review and editing. **Sophie Ping:** Conceptualization; writing – review and editing. **Kylie Rix:** Conceptualization; writing – review and editing. **Stephanie Veal:** Conceptualization; writing – review and editing. **Helen Skouteris:** Conceptualization; methodology; funding acquisition; writing – original draft; writing – review and editing; supervision.

## FUNDING INFORMATION

This project was funded through the Medical Research Future Fund (MRFF) Preventive and Public Health Research Initiative grant (APP1199826).

## CONFLICT OF INTEREST STATEMENT

None declared.

## ETHICS STATEMENT

Formal ethics approval was not sought for this study.

## Supporting information


Appendix S1

## Data Availability

The data that support the findings of this study are available from the corresponding author upon reasonable request.
