# Supplementary material for: Childhood obesity in the first 2000 days: A focus on primary health care in regional and rural Australia
Source: Aust J Rural Health. 2025 Feb 5;33(1):e13208. doi: 10.1111/ajr.13208 (PMC11795705; doi:10.1111/ajr.13208)
Supplement: Supplementary file 1 — Appendix S1 [file AJR-33-0-s001.docx]

Policies to address childhood obesity in the first 2000 days; a focus on primary healthcare in regional and rural Australia – Supplementary Material

List of Legends

[1. Literature review protocols 3](#_Toc169885682)

[Literature review 1: Improving access to primary healthcare in rural and regional areas in the first 2000 days 3](#_Toc169885683)

[Literature review 2: Models of care for delivering healthy lifestyle advice in the first 2000 days 5](#_Toc169885684)

[Literature review 3: Building partnerships with other health and social care organisations in the first 2000 days 7](#_Toc169885685)

[2. PRISMA Diagrams 10](#_Toc169885686)

[Literature review 1: Improving access to primary healthcare in rural and regional areas in the first 2000 days 10](#_Toc169885687)

[Literature review 2: Models of care for delivering healthy lifestyle advice in the first 2000 days 11](#_Toc169885688)

[Literature review 3: Building partnerships with other health and social care organisations in the first 2000 days 12](#_Toc169885689)

[3. Joanna Briggs Institute Quality Appraisals of included studies 13](#_Toc169885690)

[Literature Review 1: Improving access to primary healthcare in rural and regional areas in the first 2000 days 13](#_Toc169885691)

[Literature review 2: Models of care for delivering healthy lifestyle advice in the first 2000 days 20](#_Toc169885692)

[Literature review 3: Building partnerships with other health and social care organisations in the first 2000 days 23](#_Toc169885693)

[References 35](#_Toc169885694)

# Literature review protocols

Data S1

## Literature review 1: Improving access to primary healthcare in rural and regional areas in the first 2000 days

**Introduction**

A major impediment to the implementation of healthy lifestyle advice across the first 2000 days in rural and regional areas is access to appropriately qualified health professionals.

**Aim**

This review will aim to rapidly access and scope recently published, peer-reviewed literature to determine evidence-based methods for improving access to primary health care in rural and regional areas for pregnant women and young children (aged 0-5).

**Methods**

Population: pregnant women and young children (aged 0-5Years) living in rural and regional areas

Intervention: any

Comparison: any

Outcome: marker of increased access to health services

Time = 2017- current

Search Strategy

The major databases Medline, Global Health and PsycINFO will be searched in the OVID platform, using the search terms below:

Access* or afford* or availabilit*

AND

Primary health* OR general practi* or doctor* or physician* or medical* or nurs* or community health* or family health* or family physician* or family medic* or GP*

AND

Rural* or region* or remot*

AND

Child* OR p#ediatr* OR infan* OR neonat* OR newborn* OR preschool* OR pre school* OR toddler* OR mother* OR matern* OR baby OR babies OR pregn* OR conception OR antenatal OR antepartum OR prenatal* or pre natal* OR gestation* OR in utero OR offspring OR family OR families

Inclusion criteria:

Population: pregnant women, children aged 0-5

Intervention: any

Comparison: any

Outcome: if applicable to the study design - marker of increased access to primary health care services

Study Design: Published, peer-reviewed primary quantitative or qualitative studies, systematic reviews, policy analyses

Setting: rural and regional areas in high income countries

Time: Last five years (2017-2022)

Language: English

Exclusion criteria

Population: specific clinical groups – e.g. children with diabetes, prematurity

Outcome: outcomes other than those which provide an indication of improved health service access

Setting: exclusively major metropolitan settings, low-income countries

Study design: non-published, opinion pieces, study protocols

Time: prior to 2017

Language: non-English

Studies will be initially screened for inclusion/exclusion by title/abstract and then by full text review. This will be conducted by a senior researcher using the review software, Covidence.

Quality appraisal

Quality of included studies will be formally assessed using the Joanna Briggs Institute Quality Appraisal tool relevant to the study design.

Data Extraction

Data will be extracted on Year, Country, Aims, Design, Findings and Conclusions.

Data S2

## Literature review 2: Models of care for delivering healthy lifestyle advice in the first 2000 days

**Introduction**

Commonly cited barriers to the implementation of child obesity management in the primary care setting are a lack of knowledge and confidence at the provider level as well as a lack of time, training and resources to deliver health promotive advice.^1^ Innovative care and funding models which extend beyond the existing business model of many primary health care services are needed to facilitate the delivery of evidence-based healthy-lifestyle advice in the first 2000 days.

**Aim**

This review will rapidly access and scope recently published, peer-reviewed literature to explore models of care for delivering healthy lifestyle advice in the first 2000 days.

**Methods**

A rapid literature review will be undertaken which aims to answer the question: What models of care are effective in delivering healthy lifestyle advice in the first 2000 days?

Population: Children from conception to age 5 years, pregnant women.

Intervention: description of a care model for delivering healthy lifestyle advice in the first 2000 days

Comparison: business as usual

Outcome: marker of improved performance regarding the provision of healthy lifestyle advice in the first 2000 days (=pregnancy, children aged 0-5)

Setting: Primary health care in rural and regional areas (General practitioners, practice nurses, community health workers)

Search Strategy

The major databases Medline, Global Health and PsycINFO will be searched in the OVID platform, using the search terms below:

Primary health* OR general practi* or doctor* or physician* or medical* or nurs* or community health* or family health* or family physician* or family medic* or GP*

AND

Child* OR p#ediatr* OR infan* OR neonat* OR newborn* OR preschool* OR pre school* OR toddler* OR mother* OR matern* OR baby OR babies OR pregn* OR conception OR antenatal OR antepartum OR prenatal* or pre natal* OR gestation* OR in utero OR offspring OR family OR families

AND

Obes* OR nutrit* OR meal* OR feed* OR overweigh* OR BMI OR body mass* OR food* OR diet* OR physical* activ* OR physical* inactiv* OR sedentary OR exercis* OR weigh* OR body composition or anthropometr* OR lifestyle*

AND

region* OR rural* OR remote*

Inclusion criteria:

Population: Children from conception to age 5 years, pregnant women

Intervention: description of a care model for delivering healthy lifestyle advice in the first 2000 days – e.g. multi-disciplinary team approach, use of clinical practice guideline, other support.

Comparison: any

Outcome: any

Setting: high-income settings, rural areas

Study Design: Published, peer-reviewed primary quantitative or qualitative studies which provide an indication of the effectiveness of particular models of care to influence the behaviour/ performance of healthcare workers in terms of the delivery of health promotive advice in the first 2000 days

Time: Last five years (2017-2022)

Language: English

Exclusion criteria

Population: particular clinical subgroups – e.g. children with diabetes

Intervention:

Comparator: no comparison

Outcome: outcomes other than those which provide an indication of improved performance regarding the provision of healthy lifestyle advice in the first 2000 days.

Setting: low-income settings/countries

Study design: non-published, opinion pieces, study protocols

Time: prior to 2017

Language: non-English

Studies will be initially screened for inclusion/exclusion by title/abstract and then by full text review. This will be conducted by a senior researcher using the review software, Covidence.

Quality appraisal

Quality of included studies will be formally assessed using the Joanna Briggs Institute Quality Appraisal tool relevant to the study design.

Data Extraction

Data will be extracted on Year, Country, Aims, Design, Findings and Conclusions.

Data S3

##

## Literature review 3: Building partnerships with other health and social care organisations in the first 2000 days

Primary health care is defined as “first-contact, accessible, continued, comprehensive and coordinated patient-focused care.” ^2^ In essence, primary health care provides for the community’s health needs through addressing the broader social and environmental determinants of health and empowering individuals, families and communities to live healthy lives.^3^

However, Australian health services tend to exhibit an increasingly curative focus, operating in highly specialised, biomedical silos which are not conducive to addressing the broader health and social needs of the community, particularly for a significant and complex public health concern such as childhood obesity.

To better align with the aspirations set out in the Ottawa charter of health promotion, health services need to re-orient themselves towards a holistic, preventive approach, which considers the social, commercial and environmental determinants of health. This can be achieved through a stronger integration between health services as well as productive partnerships with broader community organisations which are responsible for the provision of care and services beyond the immediate health realm.

**Aim**

This review will aim to rapidly locate, access, and scope recently published, peer-reviewed literature to determine evidence-based methods for strengthening local partnerships between health and social care organisations to promote a more comprehensive model of primary health care in the first 2000 days.

**Methods**

A rapid literature review will be undertaken which aims to answer the question: how can primary health services partner with other health and social care organisations to promote a more comprehensive model of primary health care and improve health and wellbeing in the first 2000 days?

Population: primary health care organisations

Intervention: documented relationship with another community organisation for the purposes of improving health and wellbeing in the first 2000 days

Comparison: any

Outcome: any

Study Design: primary research (including case studies) which outline methods and outcomes of local partnerships for improving community health and wellbeing in the first 2000 days

Search Strategy

The major databases Medline, Global Health and PsycINFO will be searched in the OVID platform, using the search terms below (keyword search):

Primary health* OR general practi* or doctor* or physician* or medical* or nurs* or community health* or family health* or family physician* or family medic* or GP*

AND

relations* OR partner* or link* or collab* or integrat* or engag* or coalition* or interdisciplin* or transdisciplin* or intersector* or cross sector* or interagen*

AND

communit* OR local* OR neighbo* OR social

AND

organization* OR organisation* OR provider* OR service*

AND

Child* OR p#ediatr* OR infan* OR neonat* OR newborn* OR preschool* OR pre school* OR toddler* OR mother* OR matern* OR baby OR babies OR pregn* OR conception OR antenatal OR antepartum OR prenatal* or pre natal* OR gestation* OR in utero OR offspring OR family OR families

Inclusion criteria:

Population: primary health care organisations

Intervention: documented relationship with another community (health or non-health) organisation for the purposes of improving health and wellbeing in the first 2000 days

Comparison: any

Outcome: any

Study Design: primary research (including case studies) which outline methods and outcomes of local partnerships for improving community health and wellbeing in the first 2000 days, systematic reviews

Setting: high-income settings

Time: Last five years (2017-2022)

Language: English

Exclusion criteria

Population: Non-health related organisations only

Intervention: studies which do not describe a relationship with a community organisation (e.g. co-designed interventions with a group of end-users without a formal relationship with an established organisation), studies which look at health interventions that explicitly impact beyond the first 2000 days e.g. healthy ageing programs

Comparator:

Outcome:

Setting: low-income settings/countries

Study design: non-published, opinion pieces, study protocols

Time: prior to 2017

Language: non-English

Studies will be initially screened for inclusion/exclusion by title/abstract and then by full text review. This will be conducted by a senior researcher using the review software, Covidence.

Quality appraisal

Quality of included studies will be formally assessed using the Joanna Briggs Institute Quality Appraisal tool relevant to the study design.

Data Extraction

Data will be extracted on Year, Country, Aims, Design, Findings and Conclusions.

# 2. PRISMA Diagrams

## Literature review 1: Improving access to primary healthcare in rural and regional areas in the first 2000 days

Records removed *before screening*:

Duplicate records removed (n = 1241 )

Records identified from*:

Global Health (n = 1915 )

Medline (n = 3129 )

PsycINFO (n = 541)

**Identification**

Records excluded

(n = 4323 )

Records screened

(n = 4344 )

Reports sought for retrieval

(n = 21 )

**Screening**

Reports excluded: 14

Not primary research (n=3)

Wrong setting (n=7)

Wrong outcomes (n=2)

Not in English language (n=1)

Wrong population (n= 1)

Reports assessed for eligibility

(n = 21 )

Studies included in review

(n = 7)

**Included**

*From:*  Page MJ, McKenzie JE, Bossuyt PM, Boutron I, Hoffmann TC, Mulrow CD, et al. The PRISMA 2020 statement: an updated guideline for reporting systematic reviews. BMJ 2021;372:n71. doi: 10.1136/bmj.n71

## Literature review 2: Models of care for delivering healthy lifestyle advice in the first 2000 days

Records removed *before screening*:

Duplicate records removed (n = 1190)

Records identified from*: 6272

Global Health (n =3117 )

Medline (n=2794)

PsycINFO (n=361)

**Identification**

Records screened

(n = 5082 )

Records excluded

(n = 5064 )

**Screening**

12 records excluded:

Not a rural/regional setting (n= 5)

Low resource setting (n =3 )

Duplicate (n =1 )

Not primary research (n=1)

Did not report on provision of healthy lifestyle advice (n=2)

Full-text records assessed for eligibility

(n = 18 )

Studies included in review

(n = 6)

**Included**

## Literature review 3: Building partnerships with other health and social care organisations in the first 2000 days

Records removed *before screening*:

Duplicate records removed (n = 4547 )

Records identified from*:

Global Health (n = 4578 )

Medline (n = 7106 )

PsycINFO (n = 2779)

**Identification**

Records screened

(n = 9916 )

Records excluded**

(n = 9884 )

Reports sought for retrieval

(n = 32 )

**Screening**

Reports excluded: 20

Low income setting (n = 6 )

Wrong intervention (n = 5 )

No primary health/community partnership (n= 2 )

Not primary research (n = 3)

Wrong population (n = 2)

Not in English language (n=1)

Not focused on first 2000 days (n= 1)

Reports assessed for eligibility

(n = 32 )

Studies included in review

(n = 12)

**Included**

*From:*  Page MJ, McKenzie JE, Bossuyt PM, Boutron I, Hoffmann TC, Mulrow CD, et al. The PRISMA 2020 statement: an updated guideline for reporting systematic reviews. BMJ 2021;372:n71. doi: 10.1136/bmj.n71

# 3. Joanna Briggs Institute Quality Appraisals of included studies

## Literature Review 1: Improving access to primary healthcare in rural and regional areas in the first 2000 days

**Demirci et al, 2019^4^**

|  | Yes | No | Unclear | Not applicable |
| --- | --- | --- | --- | --- |
| Is there congruity between the stated philosophical perspective and the research methodology? | □ | □ | x | □ |
| Is there congruity between the research methodology and the research question or objectives? | x | □ | □ | □ |
| Is there congruity between the research methodology and the methods used to collect data? | x | □ | □ | □ |
| Is there congruity between the research methodology and the representation and analysis of data? | x | □ | □ | □ |
| Is there congruity between the research methodology and the interpretation of results? | x | □ | □ | □ |
| Is there a statement locating the researcher culturally or theoretically? | □ | x | □ | □ |
| Is the influence of the researcher on the research, and vice- versa, addressed? | □ | x | □ | □ |
| Are participants, and their voices, adequately represented? | x | □ | □ | □ |
| Is the research ethical according to current criteria or, for recent studies, and is there evidence of ethical approval by an appropriate body? | x | □ | □ | □ |
| Do the conclusions drawn in the research report flow from the analysis, or interpretation, of the data? | x | □ | □ | □ |

**Kapinos et al , 2019^5^**

|  | Yes | No | Unclear | Not applicable |
| --- | --- | --- | --- | --- |
| Were there clear criteria for inclusion in the case series? | x | □ | □ | □ |
| Was the condition measured in a standard, reliable way for all participants included in the case series? | x | □ | □ | □ |
| Were valid methods used for identification of the condition for all participants included in the case series? | x | □ | □ | □ |
| Did the case series have consecutive inclusion of participants? | □ | □ | x | □ |
| Did the case series have complete inclusion of participants? | □ | x | □ | □ |
| Was there clear reporting of the demographics of the participants in the study? | x | □ | □ | □ |
| Was there clear reporting of clinical information of the participants? | □ | □ | □ | x |
| Were the outcomes or follow up results of cases clearly reported? | x | □ | □ | □ |
| Was there clear reporting of the presenting site(s)/clinic(s) demographic information? | x | □ | □ | □ |
| Was statistical analysis appropriate? | x | □ | □ | □ |

**Kirby et al, 2021^6^**

|  | Yes | No | Unclear | Not applicable |
| --- | --- | --- | --- | --- |
| Is there congruity between the stated philosophical perspective and the research methodology? | x | □ | □ | □ |
| Is there congruity between the research methodology and the research question or objectives? | x | □ | □ | □ |
| Is there congruity between the research methodology and the methods used to collect data? | x | □ | □ | □ |
| Is there congruity between the research methodology and the representation and analysis of data? | x | □ | □ | □ |
| Is there congruity between the research methodology and the interpretation of results? | x | □ | □ | □ |
| Is there a statement locating the researcher culturally or theoretically? | x | □ | □ | □ |
| Is the influence of the researcher on the research, and vice- versa, addressed? | □ | x | □ | □ |
| Are participants, and their voices, adequately represented? | x | □ | □ | □ |
| Is the research ethical according to current criteria or, for recent studies, and is there evidence of ethical approval by an appropriate body? | x | □ | □ | □ |
| Do the conclusions drawn in the research report flow from the analysis, or interpretation, of the data? | x | □ | □ | □ |

**Luscombe et al, 2021^7^**

|  | Yes | No | Unclear | Not applicable |
| --- | --- | --- | --- | --- |
| Is there congruity between the stated philosophical perspective and the research methodology? | x | □ | □ | □ |
| Is there congruity between the research methodology and the research question or objectives? | x | □ | □ | □ |
| Is there congruity between the research methodology and the methods used to collect data? | x | □ | □ | □ |
| Is there congruity between the research methodology and the representation and analysis of data? | x | □ | □ | □ |
| Is there congruity between the research methodology and the interpretation of results? | x | □ | □ | □ |
| Is there a statement locating the researcher culturally or theoretically? | □ | x | □ | □ |
| Is the influence of the researcher on the research, and vice- versa, addressed? | □ | x | □ | □ |
| Are participants, and their voices, adequately represented? | x | □ | □ | □ |
| Is the research ethical according to current criteria or, for recent studies, and is there evidence of ethical approval by an appropriate body? | x | □ | □ | □ |
| Do the conclusions drawn in the research report flow from the analysis, or interpretation, of the data? | x | □ | □ | □ |

**Mathu-Muju et al, 2018^8^**

|  | Yes | No | Unclear | Not applicable |
| --- | --- | --- | --- | --- |
| Were there clear criteria for inclusion in the case series? | x | □ | □ | □ |
| Was the condition measured in a standard, reliable way for all participants included in the case series? | x | □ | □ | □ |
| Were valid methods used for identification of the condition for all participants included in the case series? | □ | □ | □ | x |
| Did the case series have consecutive inclusion of participants? | x | □ | □ | □ |
| Did the case series have complete inclusion of participants? | x | □ | □ | □ |
| Was there clear reporting of the demographics of the participants in the study? | x | □ | □ | □ |
| Was there clear reporting of clinical information of the participants? | □ | □ | □ | x |
| Were the outcomes or follow up results of cases clearly reported? | x | □ | □ | □ |
| Was there clear reporting of the presenting site(s)/clinic(s) demographic information? | x | □ | □ | □ |
| Was statistical analysis appropriate? | x | □ | □ | □ |

**Mathu-Muju et al, 2017^9^**

|  | Yes | No | Unclear | Not applicable |
| --- | --- | --- | --- | --- |
| Is there congruity between the stated philosophical perspective and the research methodology? | □ | □ | x | □ |
| Is there congruity between the research methodology and the research question or objectives? | x | □ | □ | □ |
| Is there congruity between the research methodology and the methods used to collect data? | x | □ | □ | □ |
| Is there congruity between the research methodology and the representation and analysis of data? | x | □ | □ | □ |
| Is there congruity between the research methodology and the interpretation of results? | x | □ | □ | □ |
| Is there a statement locating the researcher culturally or theoretically? | x | □ | □ | □ |
| Is the influence of the researcher on the research, and vice- versa, addressed? | x | □ | □ | □ |
| Are participants, and their voices, adequately represented? | x | □ | □ | □ |
| Is the research ethical according to current criteria or, for recent studies, and is there evidence of ethical approval by an appropriate body? | x | □ | □ | □ |
| Do the conclusions drawn in the research report flow from the analysis, or interpretation, of the data? | x | □ | □ | □ |

**Wideman et al, 2020^10^**

|  | Yes | No | Unclear | Not applicable |
| --- | --- | --- | --- | --- |
| Is there congruity between the stated philosophical perspective and the research methodology? | □ | □ | x | □ |
| Is there congruity between the research methodology and the research question or objectives? | x | □ | □ | □ |
| Is there congruity between the research methodology and the methods used to collect data? | x | □ | □ | □ |
| Is there congruity between the research methodology and the representation and analysis of data? | x | □ | □ | □ |
| Is there congruity between the research methodology and the interpretation of results? | x | □ | □ | □ |
| Is there a statement locating the researcher culturally or theoretically? | □ | x | □ | □ |
| Is the influence of the researcher on the research, and vice- versa, addressed? | □ | x | □ | □ |
| Are participants, and their voices, adequately represented? | x | □ | □ | □ |
| Is the research ethical according to current criteria or, for recent studies, and is there evidence of ethical approval by an appropriate body? | x | □ | □ | □ |
| Do the conclusions drawn in the research report flow from the analysis, or interpretation, of the data? | x | □ | □ | □ |

## Literature review 2: Models of care for delivering healthy lifestyle advice in the first 2000 days

**Ahlers-Schmidt et al, 2019^11^**

|  | Yes | No | Unclear | Not applicable |
| --- | --- | --- | --- | --- |
| Is it clear in the study what is the ‘cause’ and what is the ‘effect’ (i.e. there is no confusion about which variable comes first)? | x | □ | □ | □ |
| Were the participants included in any comparisons similar? | x | □ | □ | □ |
| Were the participants included in any comparisons receiving similar treatment/care, other than the exposure or intervention of interest? | □ | □ | □ | x |
| Was there a control group? | □ | x | □ | □ |
| Were there multiple measurements of the outcome both pre and post the intervention/exposure? | □ | x | □ | □ |
| Was follow up complete and if not, were differences between groups in terms of their follow up adequately described and analyzed? | x | □ | □ | □ |
| Were the outcomes of participants included in any comparisons measured in the same way? | x | □ | □ | □ |
| Were outcomes measured in a reliable way? | x | □ | □ | □ |
| Was appropriate statistical analysis used? | x | □ | □ | □ |

**Ekambareshwar et al, 2021^12^**

|  | Yes | No | Unclear | Not applicable |
| --- | --- | --- | --- | --- |
| Is there congruity between the stated philosophical perspective and the research methodology? | □ | □ | x | □ |
| Is there congruity between the research methodology and the research question or objectives? | x | □ | □ | □ |
| Is there congruity between the research methodology and the methods used to collect data? | x | □ | □ | □ |
| Is there congruity between the research methodology and the representation and analysis of data? | x | □ | □ | □ |
| Is there congruity between the research methodology and the interpretation of results? | x | □ | □ | □ |
| Is there a statement locating the researcher culturally or theoretically? | x | □ | □ | □ |
| Is the influence of the researcher on the research, and vice- versa, addressed? | x | □ | □ | □ |
| Are participants, and their voices, adequately represented? | x | □ | □ | □ |
| Is the research ethical according to current criteria or, for recent studies, and is there evidence of ethical approval by an appropriate body? | x | □ | □ | □ |
| Do the conclusions drawn in the research report flow from the analysis, or interpretation, of the data? | x | □ | □ | □ |

**Johnson et al, 2017^13^**

|  | Yes | No | Unclear | Not applicable |
| --- | --- | --- | --- | --- |
| Is there congruity between the stated philosophical perspective and the research methodology? | □ | □ | x | □ |
| Is there congruity between the research methodology and the research question or objectives? | x | □ | □ | □ |
| Is there congruity between the research methodology and the methods used to collect data? | x | □ | □ | □ |
| Is there congruity between the research methodology and the representation and analysis of data? | x | □ | □ | □ |
| Is there congruity between the research methodology and the interpretation of results? | x | □ | □ | □ |
| Is there a statement locating the researcher culturally or theoretically? | □ | x | □ | □ |
| Is the influence of the researcher on the research, and vice- versa, addressed? | □ | x | □ | □ |
| Are participants, and their voices, adequately represented? | x | □ | □ | □ |
| Is the research ethical according to current criteria or, for recent studies, and is there evidence of ethical approval by an appropriate body? | □ | □ | □ | x |
| Do the conclusions drawn in the research report flow from the analysis, or interpretation, of the data? | x | □ | □ | □ |

## Literature review 3: Building partnerships with other health and social care organisations in the first 2000 days

**Brown et al, 2020^14^**

|  | Yes | No | Unclear | Not applicable |
| --- | --- | --- | --- | --- |
| Were there clear criteria for inclusion in the case series? | x | □ | □ | □ |
| Was the condition measured in a standard, reliable way for all participants included in the case series? | x | □ | □ | □ |
| Were valid methods used for identification of the condition for all participants included in the case series? | □ | □ | □ | x |
| Did the case series have consecutive inclusion of participants? | x | □ | □ | □ |
| Did the case series have complete inclusion of participants? | □ | x | □ | □ |
| Was there clear reporting of the demographics of the participants in the study? | x | □ | □ | □ |
| Was there clear reporting of clinical information of the participants? | □ | □ | □ | x |
| Were the outcomes or follow up results of cases clearly reported? | x | □ | □ | □ |
| Was there clear reporting of the presenting site(s)/clinic(s) demographic information? | x | □ | □ | □ |
| Was statistical analysis appropriate? | x | □ | □ | □ |

**Corley et al, 2022^15^**

|  | Yes | No | Unclear | Not applicable |
| --- | --- | --- | --- | --- |
| Were there clear criteria for inclusion in the case series? | x | □ | □ | □ |
| Was the condition measured in a standard, reliable way for all participants included in the case series? | □ | □ | □ | x |
| Were valid methods used for identification of the condition for all participants included in the case series? | □ | □ | □ | x |
| Did the case series have consecutive inclusion of participants? | □ | □ | x | □ |
| Did the case series have complete inclusion of participants? | □ | x | □ | □ |
| Was there clear reporting of the demographics of the participants in the study? | x | □ | □ | □ |
| Was there clear reporting of clinical information of the participants? | x | □ | □ | □ |
| Were the outcomes or follow up results of cases clearly reported? | x | □ | □ | □ |
| Was there clear reporting of the presenting site(s)/clinic(s) demographic information? | □ | □ | □ | x |
| Was statistical analysis appropriate? | □ | □ | □ | x |

**Gold et al, 2018^16^**

|  | Yes | No | Unclear | Not applicable |
| --- | --- | --- | --- | --- |
| Was the sample frame appropriate to address the target population? | x | □ | □ | □ |
| Were study participants sampled in an appropriate way? | x | □ | □ | □ |
| Was the sample size adequate? | x | □ | □ | □ |
| Were the study subjects and the setting described in detail? | x | □ | □ | □ |
| Was the data analysis conducted with sufficient coverage of the identified sample? | x | □ | □ | □ |
| Were valid methods used for the identification of the condition? | x | □ | □ | □ |
| Was the condition measured in a standard, reliable way for all participants? | x | □ | □ | □ |
| Was there appropriate statistical analysis? | x | □ | □ | □ |
| Was the response rate adequate, and if not, was the low response rate managed appropriately? | □ | □ | □ | x |

**Hargreaves et al, 2017^17^**

|  | Yes | No | Unclear | Not applicable |
| --- | --- | --- | --- | --- |
| Is there congruity between the stated philosophical perspective and the research methodology? | □ | □ | □ | x |
| Is there congruity between the research methodology and the research question or objectives? | x | □ | □ | □ |
| Is there congruity between the research methodology and the methods used to collect data? | x | □ | □ | □ |
| Is there congruity between the research methodology and the representation and analysis of data? | x | □ | □ | □ |
| Is there congruity between the research methodology and the interpretation of results? | x | □ | □ | □ |
| Is there a statement locating the researcher culturally or theoretically? | □ | x | □ | □ |
| Is the influence of the researcher on the research, and vice- versa, addressed? | □ | x | □ | □ |
| Are participants, and their voices, adequately represented? | x | □ | □ | □ |
| Is the research ethical according to current criteria or, for recent studies, and is there evidence of ethical approval by an appropriate body? | x | □ | □ | □ |
| Do the conclusions drawn in the research report flow from the analysis, or interpretation, of the data? | x | □ | □ | □ |

**Kay et al, 2019^18^**

|  | Yes | No | Unclear | Not applicable |
| --- | --- | --- | --- | --- |
| Is there congruity between the stated philosophical perspective and the research methodology? | □ | □ | □ | x |
| Is there congruity between the research methodology and the research question or objectives? | x | □ | □ | □ |
| Is there congruity between the research methodology and the methods used to collect data? | x | □ | □ | □ |
| Is there congruity between the research methodology and the representation and analysis of data? | x | □ | □ | □ |
| Is there congruity between the research methodology and the interpretation of results? | x | □ | □ | □ |
| Is there a statement locating the researcher culturally or theoretically? | □ | x | □ | □ |
| Is the influence of the researcher on the research, and vice- versa, addressed? | □ | x | □ | □ |
| Are participants, and their voices, adequately represented? | x | □ | □ | □ |
| Is the research ethical according to current criteria or, for recent studies, and is there evidence of ethical approval by an appropriate body? | x | □ | □ | □ |
| Do the conclusions drawn in the research report flow from the analysis, or interpretation, of the data? | x | □ | □ | □ |

**Pawloski et al, 2022^19^**

|  | Yes | No | Unclear | Not applicable |
| --- | --- | --- | --- | --- |
| Is there congruity between the stated philosophical perspective and the research methodology? | □ | □ | □ | x |
| Is there congruity between the research methodology and the research question or objectives? | x | □ | □ | □ |
| Is there congruity between the research methodology and the methods used to collect data? | x | □ | □ | □ |
| Is there congruity between the research methodology and the representation and analysis of data? | x | □ | □ | □ |
| Is there congruity between the research methodology and the interpretation of results? | x | □ | □ | □ |
| Is there a statement locating the researcher culturally or theoretically? | □ | x | □ | □ |
| Is the influence of the researcher on the research, and vice- versa, addressed? | □ | x | □ | □ |
| Are participants, and their voices, adequately represented? | x | □ | □ | □ |
| Is the research ethical according to current criteria or, for recent studies, and is there evidence of ethical approval by an appropriate body? | x | □ | □ | □ |
| Do the conclusions drawn in the research report flow from the analysis, or interpretation, of the data? | □ | □ | □ | x |

**Olson et al, 2018^20^**

|  | Yes | No | Unclear | Not applicable |
| --- | --- | --- | --- | --- |
| Were the criteria for inclusion in the sample clearly defined? | x | □ | □ | □ |
| Were the study subjects and the setting described in detail? | x | □ | □ | □ |
| Was the exposure measured in a valid and reliable way? | x | □ | □ | □ |
| Were objective, standard criteria used for measurement of the condition? | □ | □ | □ | x |
| Were confounding factors identified? | □ | x | □ | □ |
| Were strategies to deal with confounding factors stated? | □ | x | □ | □ |
| Were the outcomes measured in a valid and reliable way? | x | □ | □ | □ |
| Was appropriate statistical analysis used? | x | □ | □ | □ |

**Rehmus et al, 2021^21^**

|  | Yes | No | Unclear | Not applicable |
| --- | --- | --- | --- | --- |
| Were patient’s demographic characteristics clearly described? | x | □ | □ | □ |
| Was the patient’s history clearly described and presented as a timeline? | □ | □ | □ | x |
| Was the current clinical condition of the patient on presentation clearly described? | □ | □ | □ | x |
| Were diagnostic tests or assessment methods and the results clearly described? | □ | □ | □ | x |
| Was the intervention(s) or treatment procedure(s) clearly described? | x | □ | □ | □ |
| Was the post-intervention clinical condition clearly described? | □ | □ | □ | x |
| Were adverse events (harms) or unanticipated events identified and described? | □ | □ | □ | x |
| Does the case report provide takeaway lessons? | x | □ | □ | □ |

**Salomonsson, 2021^22^**

|  | Yes | No | Unclear | Not applicable |
| --- | --- | --- | --- | --- |
| Were the criteria for inclusion in the sample clearly defined? | x | □ | □ | □ |
| Were the study subjects and the setting described in detail? | x | □ | □ | □ |
| Was the exposure measured in a valid and reliable way? | x | □ | □ | □ |
| Were objective, standard criteria used for measurement of the condition? | □ | □ | □ | x |
| Were confounding factors identified? | □ | x | □ | □ |
| Were strategies to deal with confounding factors stated? | □ | x | □ | □ |
| Were the outcomes measured in a valid and reliable way? | x | □ | □ | □ |
| Was appropriate statistical analysis used? | x | □ | □ | □ |

**Taveras et al, 2017^23^**

|  | Yes | No | Unclear | NA |
| --- | --- | --- | --- | --- |
| Was true randomization used for assignment of participants to treatment groups? | x | □ | □ | □ |
| Was allocation to treatment groups concealed? | x | □ | □ | □ |
| Were treatment groups similar at the baseline? | x | □ | □ | □ |
| Were participants blind to treatment assignment? | x | □ | □ | □ |
| Were those delivering treatment blind to treatment assignment? | x | □ | □ | □ |
| Were outcomes assessors blind to treatment assignment? | x | □ | □ | □ |
| Were treatment groups treated identically other than the intervention of interest? | x | □ | □ | □ |
| Was follow up complete and if not, were differences between groups in terms of their follow up adequately described and analyzed? | x | □ | □ | □ |
| Were participants analyzed in the groups to which they were randomized? | x | □ | □ | □ |
| Were outcomes measured in the same way for treatment groups? | x | □ | □ | □ |
| Were outcomes measured in a reliable way? | x | □ | □ | □ |
| Was appropriate statistical analysis used? | x | □ | □ | □ |
| Was the trial design appropriate, and any deviations from the standard RCT design (individual randomization, parallel groups) accounted for in the conduct and analysis of the trial? | x | □ | □ | □ |

**Williams et al, 2021^24^**

|  | Yes | No | Unclear | Not applicable |
| --- | --- | --- | --- | --- |
| Is there congruity between the stated philosophical perspective and the research methodology? | □ | □ | □ | x |
| Is there congruity between the research methodology and the research question or objectives? | x | □ | □ | □ |
| Is there congruity between the research methodology and the methods used to collect data? | x | □ | □ | □ |
| Is there congruity between the research methodology and the representation and analysis of data? | x | □ | □ | □ |
| Is there congruity between the research methodology and the interpretation of results? | x | □ | □ | □ |
| Is there a statement locating the researcher culturally or theoretically? | □ | x | □ | □ |
| Is the influence of the researcher on the research, and vice- versa, addressed? | □ | x | □ | □ |
| Are participants, and their voices, adequately represented? | □ | □ | □ | x |
| Is the research ethical according to current criteria or, for recent studies, and is there evidence of ethical approval by an appropriate body? | □ | x | □ | □ |
| Do the conclusions drawn in the research report flow from the analysis, or interpretation, of the data? | x | □ | □ | □ |

**Williams et al, 2021^25^**

|  | Yes | No | Unclear | Not applicable |
| --- | --- | --- | --- | --- |
| Is there congruity between the stated philosophical perspective and the research methodology? | □ | □ | □ | x |
| Is there congruity between the research methodology and the research question or objectives? | x | □ | □ | □ |
| Is there congruity between the research methodology and the methods used to collect data? | x | □ | □ | □ |
| Is there congruity between the research methodology and the representation and analysis of data? | x | □ | □ | □ |
| Is there congruity between the research methodology and the interpretation of results? | x | □ | □ | □ |
| Is there a statement locating the researcher culturally or theoretically? | □ | x | □ | □ |
| Is the influence of the researcher on the research, and vice- versa, addressed? | □ | x | □ | □ |
| Are participants, and their voices, adequately represented? | □ | □ | □ | x |
| Is the research ethical according to current criteria or, for recent studies, and is there evidence of ethical approval by an appropriate body? | x | □ | □ | □ |
| Do the conclusions drawn in the research report flow from the analysis, or interpretation, of the data? | x | □ | □ | □ |

# References

1. Rhee KE, Kessl S, Lindback S, et al. Provider views on childhood obesity management in primary care settings: a mixed methods analysis. *BMC health services research* 2018;18(1):1-10.

2. World Health Organization and the United Nations Children’s Fund (UNICEF). Operational framework for primary health care: transforming vision into action. Geneva, 2020.

3. World Health Organization. Primary health care 2022 [10 August 2022]. Available from: <https://www.who.int/health-topics/primary-health-care#tab=tab_1>.

4. Demirci J, Kotzias V, Bogen DL, et al. Telelactation via mobile app: perspectives of rural mothers, their care providers, and lactation consultants. *Telemedicine and e-Health* 2019;25(9):853-58.

5. Kapinos K, Kotzias V, Bogen D, et al. The Use of and Experiences With Telelactation Among Rural Breastfeeding Mothers: Secondary Analysis of a Randomized Controlled Trial. *J Med Internet Res* 2019;21(9):e13967. doi: 10.2196/13967 [published Online First: 2019/09/05]

6. Kirby S, Edwards K, Yu S, et al. Improving outcomes for marginalised rural families through a care navigator program. *Health Promotion Journal of Australia* 2021;32(2):285-94.

7. Luscombe GM, Hawthorn J, Wu A, et al. ‘Empowering clinicians in smaller sites’: A qualitative study of clinician's experiences with a rural Virtual Paediatric Feeding Clinic. *Australian Journal of Rural Health* 2021;29(5):742-52.

8. Mathu‐Muju KR, Kong X, Brancato C, et al. Utilization of community health workers in Canada's Children's Oral Health Initiative for indigenous communities. *Community dentistry and oral epidemiology* 2018;46(2):185-93.

9. Mathu-Muju KR, McLeod J, Donnelly L, et al. The perceptions of first nation participants in a community oral health initiative. *International journal of circumpolar health* 2017;76(1):1364960.

10. Wideman ES, Dunnigan A, Jonson‐Reid M, et al. Nurse home visitation with vulnerable families in rural areas: A qualitative case file review. *Public Health Nursing* 2020;37(2):234-42.

11. Ahlers-Schmidt CR, Schunn C, Engel M, et al. Implementation of a statewide program to promote safe sleep, breastfeeding and tobacco cessation to high risk pregnant women. *Journal of Community Health* 2019;44:185-91.

12. Ekambareshwar M, Taki S, Mihrshahi S, et al. Trial collaborators' perceptions of the process of delivering Healthy Beginnings advice via telephone calls or text messages. *Health Promotion Journal of Australia: Official Journal of Australian Association of Health Promotion Professionals* 2022;33(3):810-28.

13. Johnson R, Ansley P, Doolan-Noble F, et al. Breastfeeding peer support in rural New Zealand: the views of peer supporters. *Journal of Primary Health Care* 2017;9(2):173-77.

14. Brown J, Luderowski A, Namusisi-Riley J, et al. Can a community-led intervention offering social support and health education improve maternal health? A repeated measures evaluation of the pact project run in a socially deprived london borough. *International journal of environmental research and public health* 2020;17(8):2795.

15. Corley A, Gomes SM, Crosby LE, et al. Partnering With Faith-Based Organizations to Offer Flu Vaccination and Other Preventive Services. *Pediatrics* 2022;150(3)

16. Gold J, Tomar SL. Interdisciplinary community-based oral health program for women and children at WIC. *Maternal and Child Health Journal* 2018;22(11):1617-23.

17. Hargreaves MB, Orfield C, Honeycutt T, et al. Addressing childhood obesity through multisector collaborations: evaluation of a national quality improvement effort. *Journal of Community Health* 2017;42(4):656-63.

18. Kay EJ, Quinn C, Gude A, et al. A qualitative exploration of promoting oral health for infants in vulnerable families. *British Dental Journal* 2019;227(2):137-42.

19. Pawloski C, Hilgert J, Senturia K, et al. Medical–Dental Integration in a Rural Community Health Center: A Qualitative Program Evaluation. *Health Promotion Practice* 2022;23(3):416-24.

20. Olson T, Bowen A, Smith-Fehr J, et al. Going home with baby: innovative and comprehensive support for new mothers. *Primary Health Care Research & Development* 2019;20

21. Rehmus W, Zarbafian M, Alobaida S, et al. Integrating dermatology services into a social pediatrics network: 8 years of experience in the RICHER (Responsive, Interdisciplinary/Intersectoral, Child/Community, Health, Education and Research) program. *Pediatric Dermatology* 2021;38:174-78.

22. Salomonsson B, Kornaros K, Sandell R, et al. Short‐term psychodynamic infant–parent interventions at Child health centers: Outcomes on parental depression and infant social–emotional functioning. *Infant Mental Health Journal* 2021;42(1):109-23.

23. Taveras EM, Marshall R, Sharifi M, et al. Comparative effectiveness of clinical-community childhood obesity interventions: a randomized clinical trial. *JAMA pediatrics* 2017;171(8):e171325-e25.

24. Williams VN, Lopez CC, Tung GJ, et al. A case study of care co‐ordination between primary care providers and nurse home visitors to serve young families experiencing adversity in the Northwestern United States. *Health & Social Care in the Community* 2022;30(4):1400-11.

25. Williams VN, McManus BM, Brooks‐Russell A, et al. A qualitative study of effective collaboration among nurse home visitors, healthcare providers and community support services in the United States. *Health & Social Care in the Community* 2022;30(5):1881-93.
